# Choice Function-Based Hyper-Heuristics for Causal Discovery under Linear Structural Equation Models

**DOI:** 10.3390/biomimetics9060350

**Published:** 2024-06-10

**Authors:** Yinglong Dang, Xiaoguang Gao, Zidong Wang

**Affiliations:** School of Electronic and Information, Northwestern Polytechnical University, Xi’an 710129, China; dangyinglong@mail.nwpu.edu.cn (Y.D.); nwpu_wzd@mail.nwpu.edu.cn (Z.W.)

**Keywords:** causal discovery, structural equation model, partial correlation, hyper-heuristic

## Abstract

Causal discovery is central to human cognition, and learning directed acyclic graphs (DAGs) is its foundation. Recently, many nature-inspired meta-heuristic optimization algorithms have been proposed to serve as the basis for DAG learning. However, a single meta-heuristic algorithm requires specific domain knowledge and empirical parameter tuning and cannot guarantee good performance in all cases. Hyper-heuristics provide an alternative methodology to meta-heuristics, enabling multiple heuristic algorithms to be combined and optimized to achieve better generalization ability. In this paper, we propose a multi-population choice function hyper-heuristic to discover the causal relationships encoded in a DAG. This algorithm provides a reasonable solution for combining structural priors or possible expert knowledge with swarm intelligence. Under a linear structural equation model (SEM), we first identify the partial v-structures through partial correlation analysis as the structural priors of the next nature-inspired swarm intelligence approach. Then, through partial correlation analysis, we can limit the search space. Experimental results demonstrate the effectiveness of the proposed methods compared to the earlier state-of-the-art methods on six standard networks.

## 1. Introduction

Causal discovery from observable data is described by Judea Pearl as one of the seven important tasks and tools for moving toward a strong artificial intelligence society. It is widely used in medicine [1,2,3], biology [4], environmentology [5], and other fields. Currently, there are two types of causal modeling based on DAGs: Bayesian networks and SEMs. Bayesian networks operate on discrete data, modeling the relationships between causal variables as probabilistic relationships. In contrast, SEMs operate on continuous data, assuming the data follow a specified distribution to interpret the causal relations. So, causal discovery methods based on SEMs make it possible to guarantee the unique identification theory of causal structure. The classical SEMs proposed thus far include the linear non-Gaussian acyclic model (LiNGAM) [6], additive noise model (ANM) [7], post-nonlinear model (PNL) [8], and information-geometric causal inference (IGCI) [9].

There are two main approaches for learning a DAG: constraint-based and score-based. Constraint-based approaches, such as the well-known PC [10], utilize conditional independence (CI) tests to search for a Markov equivalence class of causal graphs and do not need to assume any kind of causal mechanism. Therefore, they can be easily extended to address more complex problems. However, high-order CI tests are time-consuming and unreliable with limited samples. Score-based approaches, which use a scoring function to estimate the quality of DAGs and then search for a DAG with the highest score, are currently the most widely utilized method. However, the number of DAGs contained in the search space increases exponentially with the number of nodes. Exact methods become infeasible because they address the entire search space, and an increasing number of heuristic methods have been proposed to address this task. Examples include K2 [11], A* [12], and GES [13], but they often become trapped in local optima. To escape local optima, nature-inspired meta-heuristic optimization algorithms have been recognized for use in DAG learning. These nature-inspired optimization algorithms include the genetic algorithm (GA) [14], evolutionary programming [15], ant colony optimization [16], cuckoo optimization [17], water cycle optimization [18], particle swarm optimization (PSO) [19,20], artificial bee colony (ABC) algorithms [21], bacterial foraging optimization (BFO) algorithms [22], and firefly algorithms (FAs) [23]. Although these optimization algorithms have achieved relatively good results, they still face the following challenges:

- As suggested by the no-free-lunch theorem, a single meta-heuristic algorithm cannot meet the different needs of various practical problems and cannot guarantee good performance in all cases.
- For large DAGs, the global search ability of the meta-heuristic algorithm is insufficient, the algorithm can easily fall into local optima, and the convergence accuracy is not high.

Hybridization of more than one meta-heuristic can make use of the differences and complementarities of each heuristic to improve the performance of DAG learning. Many recent results in the scientific literature seem to support this notion. Hybridization is the combination of different meta-heuristics or components of meta-heuristics. Unlike the hybridization of meta-heuristics, hyper-heuristics represent a hybridization approach where heuristics are used to choose or generate heuristics for solving combinatorial optimization problems. Recently, hyper-heuristics have been successfully applied to many practical problems in various fields, including the traveling salesman problem [24], the vehicle routing problem [25], the knapsack problem [26], and T-way testing [27]. According to the literature on these applications, hyper-heuristic methods increase the abstraction level of heuristic algorithms and can achieve better generalization ability so that satisfactory solutions can be obtained at a small cost. Given the excellent performance of the hyper-heuristic approach, designing hyper-heuristic algorithms is a topic worthy of study for DAG learning.

There are two main hyper-heuristic categories: heuristic selection and heuristic generation. A selection hyper-heuristic, which is the focus of our study, designs a high-level strategy to select low-level heuristics with the best performance in the search process. In this paper, we develop a hyper-heuristic with a choice function as the high-level strategy, and low-level heuristics are derived from the operators of several nature-inspired optimization algorithms. To further improve the search performance of hyper-heuristics, several common heuristic algorithm search strategies are also adopted. First, we learn from several hybrid algorithms that can reduce the size of the search space. Hybrid algorithms, such as MMHC [28] and PCS [29], are combinations of constraint-based approaches and score-based approaches. The most common strategy is to reduce the size of the search space through a constraint-based approach and then perform a search. For linear SEMs, we consider using partial correlation analysis to obtain a more compact search space, while partial v-structures are also identified as a structural prior and then integrated into the search process as an alternative or supplement to expert prior knowledge. Second, we learn multi-population strategies from swarm intelligence algorithms that enhance global search capabilities and reduce the likelihood of falling into local optima.

The main contributions of this paper are summarized as follows:

1. We propose a novel method to mine conditional independence information and determine the v-structure through partial correlation analysis, and demonstrate that this method is correct in both theory and practice. In the partial correlation analysis, two restricted search spaces are obtained, and the low-level heuristics can select the appropriate search space to improve efficiency.
2. We select the components of the existing heuristic algorithm to build the low-level algorithm library. To enhance the global search capability of large-scale DAGs, we redesign the global search operator. In addition, we design a search space switching operator for the global search operator. In the first stage, the global search operator works in the restricted search space to improve efficiency, and in the second stage, it works in the complete search space to improve accuracy.
3. We propose a multi-population choice function hyper-heuristic to provide sufficient coverage of the search space, and various groups communicate with each other through an immigration operator. To solve the problem that there is an order of magnitude difference between the fitness change and running time in DAG learning problems, we modify the choice function to balance the attention between them.

The remainder of this paper is organized as follows. In Section 2, we review related works. In Section 3, the preliminaries are introduced. In Section 4, we describe our proposed algorithm. In Section 5 and Section 6, the experiments and conclusions are presented, respectively.

## 2. Related Works

Scholars have been exploring DAG learning for more than 40 years, and Constantinou divides the research results during these years into four main research directions: ideal data, continuous optimization, weakening faithfulness, and knowledge fusion [30].

1. Ideal data. In the first direction, DAG structures are constructed using various causal discovery algorithms and optimization algorithms for datasets that are ideally unbiased and satisfy causal sufficiency and faithfulness. These algorithms are based on combinatorial optimization and form two solution directions: constraint-based approaches and score-based approaches. The specific implementation process of the constraint-based approach is divided into two steps: the first step involves conducting CI tests on variables, and the second step involves learning the global structure or local structure based on the CI test results. The most classic global structure discovery method is the PC [10] algorithm, which has the advantage of low time complexity, but at the cost of the loss of stability. Therefore, Colombo and Maatthuis et al. proposed the PC-stable [31] algorithm, which effectively eliminates the order dependence in the process of skeleton determination and edge orientation. It is also a constraint-based algorithm widely recognized and used by scholars in recent years, and it is used as a comparison algorithm in this paper. In addition, Spirtes et al. proposed the FCI [10] algorithm in response to the existence of hidden variables or confusion factors in research questions. Some scholars have improved it in recent years, such as RFCI [32] and FCI+ [30]. The local structure discovery method focuses on learning Markov blankets (MBs) in a DAG. The best-known method for local structure discovery is IAMB [33], which uses conditional mutual information to determine the order in which individual variables are incorporated into the MB. Later, improved versions were proposed: Inter-IAMB and Fast-IAMB. In addition, Tsamardios and Aliferis et al. proposed the MMPC, HITON-PC, and SI-HITON-PC [30] algorithms to discover MBs. Note that the performance of constraint-based approaches, which employ statistical tools to test conditional independence in the empirical joint distribution, may be severely limited by the hypothesis tests they use. Score-based approaches can be divided into approximate approaches and exact approaches according to whether they can obtain the global optimal solution. With well-defined scores, such as the Bayesian Information Criterion (BIC), the Minimum Description Length (MDL), and the Bayesian Dirichlet equivalence (BDe), score-based approaches turn causal discovery problems into combinatorial optimization problems. Based on this, several exact methods for solving combinatorial optimization problems, such as dynamic programming [34], branch-and-bound [35], and integer linear programming [36], have been applied to DAG learning. Due to the poor scalability of exact approaches, approximate approaches have gained extreme popularity. HC [37], which uses a greedy strategy and different operators to search the neighborhood of the current DAG and update the optimal structure until the termination condition is reached, is the most classical approximate learning algorithm in DAG space. However, this algorithm can easily fall into local optima. Therefore, many nature-inspired optimization algorithms have emerged in recent years, among which PSO [20], ABC [21], and GAs [14] are widely used meta-heuristic algorithms, and many versions of these algorithms have been proposed. Although these meta-heuristic algorithms have achieved relatively good results, their search and generalization abilities still need to be improved. Therefore, this paper adopts a hyper-heuristic approach to DAG learning to obtain stronger search and generalization abilities compared to a single heuristic approach. To the best of our knowledge, hyper-heuristic methods have not been applied to DAG learning.

2. Continuous optimization. Most of the causal structures output by traditional constraint-based approaches and score-based approaches belong to the Markov equivalence class. To solve the problem of the Markov equivalence class, the method of introducing SEMs into causal models is receiving increasing attention. In SEMs, if some additional assumptions are made about the functional and/or parametric forms of the underlying true data-generating structure, then one can exploit asymmetries to identify the direction of causality. For example, Shimizu et al. [6,38] first proposed an estimation method based on independent component analysis (ICA) for LiNGAM, which is unique enough to identify the complete DAG by the non-Gaussian properties of the data. For nonlinear data, Hoyer et al. [7] proposed the ANM to infer causality based on the assumption of independence between cause variables and noise variables. Compared with the ANM, Zhang et al. [8] proposed PNL, which describes the data generation process more generally. Janzing et al. [9] started from the perspective of information geometry and made causal inferences based on information entropy. In 2018, Zheng et al. first proposed NOTEARS [39], which formulates the structure learning problem as a continuous optimization problem by introducing a smooth characterization of acyclicity. This method makes it possible to use gradient updating to acquire large-scale learning and online learning abilities. On this basis, an increasing number of machine learning methods, such as neural networks [40], reinforcement learning [41], and autoencoders [42], have been introduced into this field. In addition, some updated versions of NOTEARS have also been proposed recently, such as NO TEARS+ [43], NO BEARS [44], and NO FEARS [45]. However, NOTEARS and its variants still lack a theoretical analysis of the unique identification of this model [46]. Moreover, in our experiments, NOTEARS sometimes failed to return a DAG.

3. Weakening faithfulness. Traditional causal faithfulness is a very demanding requirement, and theorists are constantly trying to relax the use of faithfulness “bottom lines” regarding data distribution and independence tests to improve the robustness of models by using more relaxed faithfulness. Unlike the PC algorithm, which is based on complete causal faithfulness, the CPC [47] algorithm uses weaker adjacency faithfulness and directed faithfulness in the v-structure determination phase. Zhang and Spirtes believe that this weak faithfulness hypothesis can also be applied in the skeleton determination stage, so triangular faithfulness has been proposed [48]. In addition, Cheng et al. proposed the TPDA, which requires a stronger faithfulness hypothesis (monotone faithfulness).

4. Knowledge fusion. Expert knowledge is often used to assist in DAG modeling, and the integration of expert knowledge is divided into two methods: soft constraints and hard constraints. The former guides or intervenes in the learning process, while the latter forces the final learning outcome to meet certain conditions. For hard constraints, De Campos and Castellano et al. took the lead in modifying HC and PC algorithms to make the learning results meet the given edge constraints [49]. Later, De Campos proposed an improved B&B algorithm [50], which supports predetermination of the direction of partial edges before learning. Borboudakis and Tsarmadios first proposed applying this constraint to PC and FCI algorithms to improve the accuracy of the edge orientation phase [51]. For soft constraints, different initial search graphs or restricted search spaces can also be regarded as soft constraints of score search algorithms, such as MMHC [28] and PC-PSO [19]. In our algorithm, partial correlation is used to mine structural priors as a supplement or alternative for expert knowledge to guide the search process.

In summary, we consider introducing a hyper-heuristic method guided by expert knowledge to improve the search performance of causal discovery algorithms.

## 3. Background

### 3.1. DAG Model

A graph G=(V,E) represents a joint distribution $P_{X}$ as a factorization of *n* variables $X=\left\{X_{1},\cdots ,X_{n}\right\}$, using *n* corresponding nodes v∈V and connecting edges (i,j)∈E, where (i,j) indicates an edge between $v_{i}$ and $v_{j}$. If all the edges are directed and there are no cycles, we have what is known as a DAG.

**Definition** **1** (v-structure)**.** *In a DAG, if there are two distinct adjacent nodes of X on a simple path, and both of them are parents of X, then these three nodes form a v-structure, and node X is called a collider. Otherwise, we call X a noncollider [37].*

**Definition** **2** (d-separation)**.** *Two nodes $X_{i},X_{j}$ are d-separated by Z⊆$X\setminus \left\{X_{i},X_{j}\right\}$ if every simple path from $X_{i}$ to $X_{j}$ is blocked by Z. Note that a simple path is blocked if there is at least one noncollider in Z or if at least one collider and all its descendants are not in Z [37].*

### 3.2. SEMs and Partial Correlation

An SEM is a set of equations describing the value of each node $X_{i}$ in *X* as a function $f_{X}$ of its parents $pa\left(X_{i}\right)$ and a random disturbance term $u_{X_{i}}$:

(1) $$x_{i}=f_{X_{i}}\left(pa\left(X_{i}\right),u_{X_{i}}\right)$$

where the functions $f_{X_{i}}$ can be defined as linear or nonlinear. If we restrict it to be linear, we set the formula of a linear SEM as follows:

(2) $$x_{i}=w_{X_{i}}^{T}pa\left(X_{i}\right)+u_{X_{i}}$$

**Definition** **3** (Partial correlation)**.** *The partial correlation coefficient between two nodes $X_{i},X_{j}\in X$, given a set of conditions $Z\subseteq X\setminus \left\{X_{i},X_{j}\right\},$ denoted as $\rho \left(X_{i},X_{j}|Z\right),$ or simply $\rho_{ij},$ is the correlation of the residuals $R_{X_{i}}$ and $R_{X_{j}}$ resulting from the least-squares linear regression of $X_{i}$ on Z and $X_{j}$ on Z, respectively [29].*

The most common method for calculating the partial correlation coefficient relies on inverting the correlation matrix *R* of *X*. Given $R^{-1}=\left(r_{ij}\right),$ the partial correlation coefficient can be efficiently computed according to Equation (3).

(3) $$\rho \left(X_{i},X_{j}|Z\right)=-r_{ij}/\sqrt{r_{ii}r_{jj}}$$

In particular, the full partial correlation between two nodes $X_{i},X_{j}$ means that the set of conditions *Z* is equal to $X\setminus \left\{X_{i},X_{j}\right\},$ and the set of conditions *Z* corresponding to a local partial correlation is a possible subset of $X\setminus \left\{X_{i},X_{j}\right\}.$

**Theorem** **1.** *When the data are generated by linear SEMs, if the random disturbance term $u_{X_{i}}$ has constant variance and is uncorrelated, the partial correlation analysis can be used as a criterion in the CI test [52].*

**Theorem** **2.** *If the sample size (denoted as m) of a given dataset generated by linear SEMs is sufficiently large (m>120), the test statistic t concerning the partial correlation coefficient approximately follows a t-distribution with m−n degrees of freedom [29].*

(4) $$t=\frac{\rho_{ij}}{\sqrt{\left(1-\rho_{ij}^{2}\right)/\left(m-n\right)}}∼t_{m-n}$$

**Definition** **4** (Bayes factor)**.** *Given a dataset D, the Bayes factor for a null hypothesis $H_{0}$ over an alternative hypothesis $H_{1}$, denoted as $BF_{01},$ can be written according to Equation (5).*

(5) $$BF_{01}=\frac{P\left(D|H_{0}\right)}{P\left(D|H_{1}\right)}$$

For partial correlation analysis, the Bayes factor provides an index of preference for one hypothesis over another that is more intuitive in interpretation than the traditional *p* value. Since *p* values are often misunderstood and misused, the Bayes factor is used as a significance test for partial correlation analysis in this paper [53]. The reason that the Bayes factor is not commonly used is that it is inconvenient to calculate. In this paper, because the partial correlation coefficient approximately follows a *t*-distribution, the Bayes factor can be directly computed using an approximation algorithm.

(6) $$BF_{01}\approx \sqrt{n{\left(1+\frac{t^{2}}{m-n}\right)}^{-n}}$$

### 3.3. Scoring Function

The scoring function used in this paper is the BIC, which is composed of the goodness of fit of a model and the penalty for model complexity. The BIC score is defined as:

(7) $${Score}_{BIC}=\sum_{j=1}^{n}\left(NLL\left(X_{j},pa\left(X_{j}\right),{\hat{\theta }}_{j}^{mle}\right)+\frac{\left|{\hat{\theta }}_{j}^{mle}\right|}{2}\log m\right)$$

where $\sum_{j=1}^{n}NLL\left(X_{j},pa\left(X_{j}\right),{\hat{\theta }}^{mle}\right)$ denotes the negative log-likelihood used to evaluate the goodness of fit of a model, $\sum_{j=1}^{n}\frac{\left|{\hat{\theta }}_{j}^{mle}\right|}{2}$ logm denotes the penalty for model complexity, ${\hat{\theta }}_{j}^{mle}$ denotes the maximum likelihood estimate of the parameters for node $X_{j},$ and $\left|{\hat{\theta }}_{j}^{mle}\right|$ is the number of estimated parameters for node $X_{j}$, which is equal to the number of its parents.

**Theorem** **3.** *In a linear SEM, the best linear unbiased estimator of the parameters is the ordinary least-squares estimator if the random disturbance term $u_{X_{i}}$ has a mean of zero and constant variance and is uncorrelated.*

In this paper, the least-squares method was used for parameter estimation. It is a statistical method used to determine the best-fit line by minimizing the sum of squares created by a mathematical function. Therefore, the negative log-likelihood for node $X_{j}$ can be computed according to Equation (8),

(8) $$NLL\left(X_{j},pa\left(X_{j}\right),{\hat{\theta }}_{j}^{mle}\right)=\sum_{j=1}^{m}{\left(x_{ij}-{\left({\hat{\theta }}_{j}^{mle}\right)}^{T}pa\left(x_{ij}\right)\right)}^{2}$$

where ${\hat{\theta }}_{j}^{mle}$ is equal to the parameter estimated by the least-squares method and can be computed according to Equation (9),

(9) $${\hat{\theta }}_{j}^{mle}={\left(x^{\prime }x\right)}^{-1}x^{\prime }x_{j}$$

where $x_{j}$ denotes the vector of observations on $X_{j}$ and *x* denotes the vector of observations on its parents.

## 4. Methodology

In this section, we first propose a new method called structural priors by partial correlation (SPPC), where the key idea is to use partial correlation analysis to mine conditional independence information. Next, this conditional independence information is integrated into the hyper-heuristic algorithm as a structural prior.

### 4.1. SPPC

Due to the equivalence of zero partial correlation and CI for linear SEMs, the goal of the SPPC algorithm is to use partial correlation analysis to narrow the search space as much as possible in addition to identifying partial v-structures. The SPPC algorithm starts with an empty graph and consists of three stages: full partial correlation, local partial correlation, and identification of v-structures. Through these three stages, we can obtain the global search space (GSS), local search space (LSS), and v-structure (V). The pseudocode is shown in Algorithm 1, and we explain each stage in more detail in the following paragraphs. The three stages can be summarized as follows:

1. For any two nodes $X_{i},X_{j},$ add an edge $X_{i}-X_{j}$ if the full partial correlation coefficient is significantly different from zero.
2. For every edge $X_{i}-X_{j}$ in the undirected graph built in step 1, we perform a local partial correlation analysis that looks for a d-separating set Z. If the partial correlation $\rho \left(X_{i},X_{j}|Z\right)$ vanishes, we consider this edge to be a spurious link caused by v-structure effects and then remove it.
3. For every edge $X_{i}-X_{j}$ that is removed in step 2, we find the colliders contained in *Z*. If node *U* is a collider, we add two edges $X_{i}\to$$U,X_{j}\to U.$

**Algorithm 1:** SPPC 
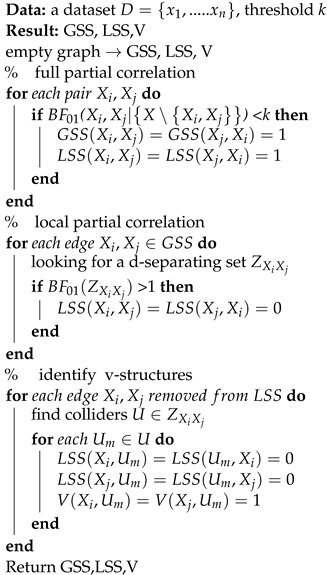


In the first stage, we perform a full partial correlation analysis and reconstruct a Markov random field. In the full partial correlation analysis, if $BF_{01}\left(X_{i},X_{j}\right)$ is less than a threshold k, we consider that the two nodes are correlative and connect with each other in the GSS and LSS. In contrast, if $BF_{01}\left(X_{i},X_{j}\right)$ is greater than the threshold k, we consider that the two nodes are uncorrelated. If the data satisfy the faithfulness assumption, the GSS derived from the identified undirected graph may resemble a moral graph. Therefore, we treat the GSS as the primary search space to ensure the completeness of the search space. Unfortunately, in the GSS, all parents of colliders are connected, and the v-structures are transformed into triangles. It should be noted that these spurious links caused by v-structure effects have a more severe negative impact on the search process compared to other error edges. When dealing with large-scale problems, the GSS cannot effectively alleviate the inefficiency of the search algorithm, and it easily falls into local optima. Fortunately, the partial correlation coefficient for the CI test is easy to calculate, even when the size of the condition set is large. Therefore, to improve search efficiency, we consider further mining conditional independence information in the second stage.

In the second stage, our goal is to find a set *Z* that blocks all simple paths between two nodes $X_{i},X_{j}.$ Obviously, the exhaustive method is inefficient and undesirable. Therefore, heuristic strategies are usually used to find such a cut set. For example, a two-phase algorithm [54] utilized a heuristic method based on monotone faithfulness that employs the absolute value of partial correlation as a decision criterion. However, monotone faithfulness is sometimes a bad assumption [55]. In this paper, we propose a new heuristic strategy to determine a d-separating set, and the pseudocode is shown in Algorithm 2. To illustrate how our heuristic strategy works, some relevant concepts are briefly introduced.
**Algorithm 2:** Local partial correlation 
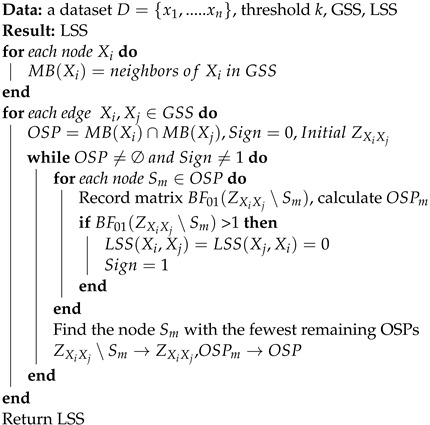

**Theorem** **4.** *For any two nodes $X_{i},X_{j},$ if there is no edge between them, we can determine a d-separating set by choosing nodes only from either $MB\left(X_{i}\right)$ or $MB\left(X_{j}\right)$ [52]. Here, $MB\left(X_{i}\right)$ denotes the Markov random field of nodes $X_{i}$.*

This theorem enables us to perform local partial correlation analysis on small sets, which makes the estimation results more efficient and stable. Next, the most important task of a heuristic strategy is to find an appropriate metric to tightly connect the CI test with d-separation.

**Definition** **5** (Simple path)**.** *A simple path is an adjacency path that does not contain duplicate nodes [52].*

According to the definition of d-separation, for any two nodes $X_{i},X_{j},$ if there exists such a cut set *Z* that makes the two nodes conditionally independent, we can finally find it by blocking all the simple paths between the two nodes.

**Definition** **6** (Active path)**.** *For any two nodes $X_{i},X_{j},$ given a simple path U between the two nodes, the path U is blocked by Z if and only if at least one noncollider on U is in Z or at least one collider and all of its descendants are not in Z [52]. If path U is not blocked by Z, we call path U an active path on Z.*

**Definition** **7** (Open simple path)**.** *For any two nodes $X_{i},X_{j},$ and given a set of conditions Z, for a node $Z_{m}$ in Z, if $\rho \left(X_{i},Z_{m}|Z\cup X_{j}\setminus \left\{Z_{m}\right\}\right)$ and $\rho \left(X_{j},Z_{m}|Z\cup X_{i}\setminus \left\{Z_{m}\right\}\right)$ are both significantly different from zero, we refer to the simple paths from $Z_{m}$ to $X_{i}$ and $X_{j}$ as open. In this case, $Z_{m}$ is said to have an open simple path (OSP) to $X_{i},X_{j}$ on Z.*

Notably, an OSP is different from an active path in a directed graph because we cannot determine whether node $Z_{m}$ is a noncollider or a collider. The initial OSP is the intersection of the Markov random fields of two nodes.

**Theorem** **5.** *For any two nodes $X_{i},X_{j},$ given a set of conditions Z, if there is no edge between the two nodes in the underlying graph and $\rho \left(X_{i},X_{j}|Z\right)$ is significantly different from zero, then all active paths on Z with colliders must satisfy that every collider and all of its descendants contain a node (denoted as $\left.Z_{m}\right)$ that belongs to Z and has an OSP to $X_{i},X_{j}.$*

**Proof** **of** **Theorem** **5.** If $\rho \left(X_{i},X_{j}|Z\right)$ is significantly different from zero, there must be at least one active path between $X_{i}$ and $X_{j}$ on Z, denoted as set U. For any path in U, denoted as path u, according to the definition of the active path, we know that all the noncolliders on *u* are not in *Z* and every collider satisfies that itself or at least one of its descendants is in Z. For any collider in u, let $Z_{m}$ denote the node that satisfies the above condition. We can easily construct an active path based on path *u* between $Z_{m}$ and $X_{i}$ on $Z\cup X_{j}\setminus \left\{Z_{m}\right\}$, and similarly between $Z_{m}$ and $X_{j}.$ Therefore, we can consider that $\rho \left(X_{i},Z_{m}|Z\cup X_{j}\setminus \left\{Z_{m}\right\}\right)$ and $\rho \left(X_{j},Z_{m}|Z\cup X_{i}\setminus \left\{Z_{m}\right\}\right)$ are both significantly different from zero, and then $Z_{m}$ has an OSP to $X_{i},X_{j}.$    □

If path *u* does not contain a collider, we cannot block this path by removing nodes. Therefore, our heuristic strategy is to start with an initial set that contains the d-separation set and then block the simple paths by gradually removing nodes that have OSPs to $X_{i},X_{j}.$ Throughout the process, as each node with an OSP is removed, we observe the number of remaining nodes that have OSPs, and this number is used as a criterion to determine which node to delete. In this paper, we greedily choose the node with the lowest value for removal. When no node in the conditional set has an OSP, the search stops.

In the third stage, our task is to orient some edges correctly by detecting v-structures. For each edge removed in the local partial correlation analysis, we find the colliders contained in Z. If node *U* is a collider, we add two edges to V.

### 4.2. Proposed Multi-Population Choice Function Hyper-Heuristic

**Figure 1:**
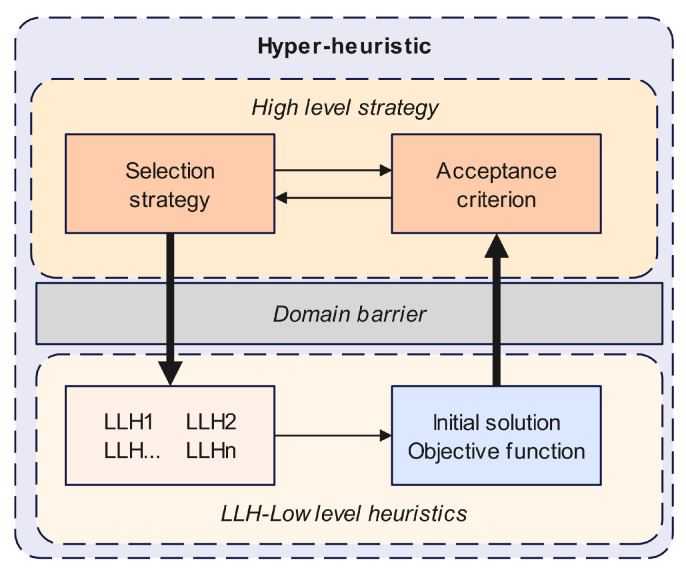
Generic structure of a traditional hyper-heuristic model.

Hyper-heuristics are high-level methodologies that perform a search over the space formed by a set of low-level heuristics when solving optimization problems. In general, a hyper-heuristic contains two levels—a high-level strategy and low-level heuristics—and there is a domain barrier between the two. The former comprises two main stages: a heuristic selection strategy and a move acceptance criterion. The latter involves a pool of low-level heuristics, the initial solution, and the objective function (often also the fitness or cost function). The working principle of hyper-heuristics is shown in Figure 1.

#### 4.2.1. The High-Level Strategy

Various combinations of heuristic selection strategies and move acceptance criteria have been reported in the literature. Classical heuristic selection strategies include choice functions, nature-inspired algorithms, multi-armed bandit (MAB)-based selection, and reinforcement learning, while move acceptance criteria include only improvement, all moves, simulated annealing, and late acceptance. In this article, we use the “choice function accept all moves” as a high-level strategy, which evaluates the performance score (F) of each LLH using three different measurements: $f_{1},f_{2},$ and $f_{3}.$ The specific calculation method is shown in Equation (10):

(10) $$F\left(H_{i}\right)=\varphi f_{1}\left(H_{i}\right)+\varphi f_{2}\left(H_{i},H_{j}\right)+\delta f_{3}\left(H_{i}\right)$$

Parameter $f_{1}$ reflects the previous performance of the currently selected heuristics, $H_{i}.$ The value of $f_{1}$ is evaluated using Equation (11),

(11) $$f_{1}\left(H_{i}\right)=I\left(H_{i}\right)/T\left(H_{i}\right)+\varphi f_{1}\left(H_{i}\right)$$

where $I\left(H_{i}\right)$ is the change in solution quality by $H_{i}$ and is set to 0 when the solution quality does not improve. $T\left(H_{i}\right)$ is the time taken by $H_{i}.$

Parameter $f_{2}$ attempts to capture any pairwise dependencies between heuristics. The values of $f_{2}$ are calculated for the current heuristic $H_{i}$ when employed immediately following $H_{j}$, using Equation (12),

(12) $$f_{2}\left(H_{i},H_{j}\right)=I\left(H_{i},H_{j}\right)/T\left(H_{i},H_{j}\right)+\varphi f_{2}\left(H_{i},H_{j}\right)$$

where $I\left(H_{i},H_{j}\right)$ is the change in solution fitness and $T\left(H_{i},H_{j}\right)$ is the time taken by both the heuristics. Similarly, $I\left(H_{i},H_{j}\right)$ is set to 0 when the solution does not improve.

Parameter $f_{3}$ captures the time elapsed since the heuristic $H_{i}$ was last selected. The value of $f_{3}$ is evaluated using Equation (13):

(13) $$f_{3}\left(H_{i}\right)=\tau \left(H_{i}\right)$$

The value range of parameters φ and δ is (0,1) and is initially set to 0.5. If the solution quality improves, φ is rewarded heavily by being assigned the highest value (0.99), whereas it is harshly punished by being assigned the lowest value (0.01). If the solution quality deteriorates, φ decreases linearly, and δ increases by the same amount. The values of both parameters are calculated using Equations (14) and (15):

(14) $$\varphi_{t}=\left\{\begin{array}{cc} 0.99, & \;\mathrm{if}\;\mathrm{quality}\;\mathrm{improves}\;\\ \max\left\{\varphi_{t-1}-0.01,0.01\right\}, & \;\mathrm{if}\;\mathrm{quality}\;\mathrm{deteriorates}\; \end{array}\right.$$

(15) $$\delta_{t}=1-\varphi_{t}$$

For each LLH, the respective values of *F* are computed using the same parameters φ and δ. The setting scheme of these two weight parameters makes the intensification component the dominating factor in the calculation of *F* while ensuring the diversification of the heuristic search process. However, in DAG learning problems, there is usually an order of magnitude difference between the fitness change and running time. As a result, the balance between the intensification component and the diversification cannot be guaranteed. To solve this problem, we record the values of $I\left(H_{i}\right)/T\left(H_{i}\right)$ and $I\left(H_{i},H_{j}\right)/T\left(H_{i},H_{j}\right)$ when the current heuristic increases the score of the optimal structure. Then, all the previously recorded values are linearly transformed into the interval (a∗m,b∗m), where m represents the average running time of all the calls. Coefficients a and b are used to balance the fitness change and running time, taking values of 0.1 and 0.2, respectively, in this article.

#### 4.2.2. The Low-Level Heuristics

In this section, we introduce the 13 operators that make up the low-level algorithm library, which is primarily derived from several nature-inspired meta-heuristic optimization algorithms. For example, we decompose the BNC-PSO algorithm into three operators: the mutation operator, cognitive personal operator, and cooperative global operator. In addition, we modify the three operators. First, the mutation operator works on the GSS to improve its efficiency. Second, the acceleration coefficient of the cooperative global operator increases linearly from 0.1 to 0.5 to avoid prematurity.

For the BFO algorithm, we choose only two operators: the chemotactic operator and the elimination and dispersal operator. Addition, deletion, and reversion operators are three candidate directions for each bacterium to select in the chemotactic process, and for large DAG learning, these local operations can be blind and inefficient. Therefore, we consider these three operations to be used only to manipulate the parent set of a node. Specifically, the addition operation continuously adds possible parents to the selected node to improve the score, and its search space is the GSS. Correspondingly, the deletion operation and the reversion operation perform sequential deletion or parent–child transformation of the parent set of the selected node to improve the score. The elimination and dispersal operator is a global search operator, and we need to redesign a restart scheme only for the parent set of a selected node. For a selected node, we perform a local restart of the optimal structure in the population as a bacterial elimination and dispersal operation. First, we remove all the parent nodes of the selected node, calculate the score at this point, and record the structure at this point as the starting point for the restart. Second, in the search space, an addition chemotaxis operation is performed on the selected node to find a potential parent set, which is subsequently sorted by partial correlation values. Note that we are not updating the starting point structure in this step. Third, we add the nodes of the potential parent set one by one to the selected node, and if a node can improve the score, we add both itself and its parent and update the structure. Fourth, for the parent nodes that have been added, we greedily remove the one that has the greatest negative impact on the score and update the structure until the score cannot be improved. The nodes that have the greatest negative impact are achieved by the deletion chemotactic operation. Finally, we perform a reversion operation. The startup of the elimination and dispersal operator is controlled by the parameter $c_{3}$, which increases linearly from 0.1 to 1 and is computed using Equation (16),

(16) $$c_{3}=0.1+0.9\left(L/L_{\max}\right)$$

where *L* represents the number of iterations in which the global maximum score did not improve, and $L_{\max}$ represents the maximum number of iterations allowed without increasing the global maximum score.

We decompose the ABC algorithm into three operators: worker bees, onlooker bees, and scout bees. Worker bees and onlooker bees, as local search operators, continue to work on the GSS. We redesign the scout bees to accommodate large-scale DAG learning. For a selected node, we perform a local restart of the optimal structure in the population. First, we record the parent set of the selected node. Second, a parent node is selected, and a parent–child transformation is performed with the selected node. Third, the addition, deletion, and reversion chemotaxis operations are performed successively. If the score of the new structure is higher than the score of the optimal structure, the structure is updated as a new starting point. Finally, we skip to step 2 and continue until all parent nodes have been tested. The startup of the scout is controlled by the parameter $l_{m}$ when the individual best score does not improve for $l_{m}$ consecutive iterations.

Inspired by the moth–flame optimization algorithm, we randomly arrange the individual historical optimal solutions as flames and design moths to fly around them, which is equivalent to moths learning from the flames. The learning mode is the same as that of the BNC-PSO algorithm. Similarly, we adopt the learner phase from the teaching–learning-based optimization algorithm. In the current generation, each student is randomly assigned a collaborator to learn from if they are better than themselves, with the learning mode aligned with that of the BNC-PSO algorithm.

To make efficient use of structural priors, expert knowledge operators are designed. In this operator, a fixed proportion of individuals are selected to be guided by expert knowledge or a structural prior, i.e., all identified v-structures are given. For large-scale DAG learning, an insufficient sample size often leads to overfitting problems. To reduce the complexity of the model, pruning operators are designed to remove all edges if the score change caused by these edges is less than a threshold μ. This threshold is shared by all operators as the basis for judging whether the score has improved. In addition, a more efficient neighborhood perturbation operator is designed to operate on the LSS.

#### 4.2.3. Framework of Our Algorithm

In this section, we describe the workflow of our proposed multi-population choice function hyper-heuristic (MCFHH) algorithm, the framework of which is shown in Algorithm 3. The MCFHH algorithm starts by randomly generating the initial valid population, and the initial valid population is obtained by performing several local hill-climbing operations on V. Next, we divide the population evenly into several groups, and each group runs its own choice function individually. Our algorithm terminates when the optimal score does not improve in successive $L_{\max}$ generations or the maximum number of allowed iterations is reached. In addition, we introduce the migration operator and search space switching operator when running the algorithm.

The migration operator runs only after a certain number of iterations, and we set it to a minimum value between 100 and N. In the migration operation, we record the optimal structure of each subgroup and then swap the best with the worst. To avoid inbreeding, we use the inbreeding rate as a parameter to limit immigration operations. For DAG learning, we measure the inbreeding rate using the Hamming distance between the optimal individual of each subgroup and the globally optimal individual. In this paper, if the Hamming distance is less than 4, we assume that the optimal individual of the subgroup and the globally optimal individual are close relatives. The inbreeding rate is defined as the number of close relatives of a globally optimal individual divided by the number of subgroups, which, in this paper, is limited to no more than 0.6.

For large-scale DAG learning, the GSS cannot guarantee the completeness of the search space when the sample size is insufficient. Therefore, we introduce a search space switching operator. The search space switching operator is executed only once when the number of iterations without an increase in the highest score reaches $L_{\max}.$ After execution, all global search operators operate within the complete search space (CSS) to correct errors caused by possible incompleteness in the GSS, and the number of iterations without an increase in the highest score is recalculated. This switching scheme is a balanced strategy that can improve efficiency in the early stage of the algorithm and improve accuracy in the late stage of the algorithm.
**Algorithm 3:** MCFHH 
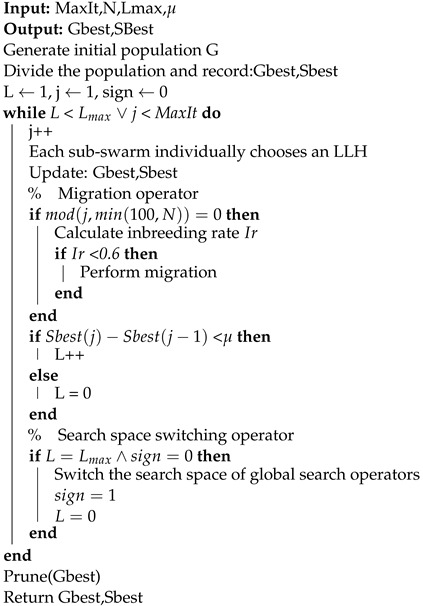


## 5. Experiments

In this section, several existing competitive algorithms and networks are selected to test the performance of the MCFHH algorithm. The following algorithms were selected for comparison: the PC-stable, LiNGAM, PCS, BNC-PSO, and NOTEARS algorithms (https://github.com/xunzheng/notears, accessed on 16 May 2024). We added structural prior knowledge, including the initial population and the GSS, to the BNC-PSO algorithm in this paper. All the experiments were implemented and executed on a computer running Windows 10 with an AMD 1.7 GHz CPU and 16 GB of memory. NOTEARS was implemented in Python 3.10.5, and the other algorithms were implemented in MATLAB R2020a.

### 5.1. Networks and Datasets

In our experiments, six networks were selected from the BNLEARN repository (https://www.bnlearn.com/bnrepository/, accessed on 16 May 2024), and a summary of these networks is shown in Table 1.

The datasets used in the experiments were generated by linear SEMs. Three different SEMs were designed, including the linear Gaussian model and the linear non-Gaussian model, as follows:

(17) $$\begin{array}{c} \left(1\right)x_{i}=w_{1X_{i}}^{T}pa\left(X_{i}\right)+N\left(0,1\right)\\ \left(2\right)x_{i}=w_{2X_{i}}^{T}pa\left(X_{i}\right)+N\left(0,1\right)\\ \left(3\right)x_{i}=w_{1X_{i}}^{T}pa\left(X_{i}\right)+\mathrm{rand}\left(-1,1\right) \end{array}$$

where $w_{1X_{i}}=\pm 1+N\left(0,1\right)/4$ and $w_{2X_{i}}=\mathrm{rand}\left(0.2,1\right).$ For SEM1, the weight $w_{1X_{i}}$ is a Gaussian distribution, and the random disturbance term is also a Gaussian distribution. Thus, SEM1 follows a multivariate Gaussian distribution and is a linear Gaussian model. For SEM2, the weight $w_{2X_{i}}$ is randomly and uniformly distributed, and the random disturbance term follows a Gaussian distribution. Thus, SEM2 also follows a multivariate Gaussian distribution and is a linear Gaussian model. For SEM3, the weight is a Gaussian distribution, and the random disturbance term is randomly and uniformly distributed. Thus, SEM3 is a linear non-Gaussian model.

### 5.2. Performance Evaluation of the MCFHH Algorithm

The parameters of the MCFHH algorithm are listed in Table 2, and the parameters of the other algorithms are the best values from the corresponding literature. To evaluate the performance of these algorithms, the following metrics were used:

- BIC: the BIC score of the final output structure.
- SBS: the BIC score of the standard network.
- AD: the difference of arcs incorrectly added over all trials.
- DD: the difference of arcs incorrectly deleted over all trials.
- RD: the difference of arcs incorrectly reversed over all trials.
- RET: the execution time of the restriction phase.
- SET: the execution time of the search phase.
- F1: the F1 score of the final output structure.

The first performance metric is the BIC (higher is better), representing the score of the final output structure. The calculation method of the BIC was introduced in Section 3.3. The SBS represents the score of the original network, which is a fixed reference value based on the sample data. AD, DD, and RD are used to evaluate the structural errors of the learning result, representing the number of incorrectly added edges, incorrectly deleted edges, and incorrectly reversed edges, respectively, in the final output network compared to the original network. RET and SET represent the execution times of the restriction phase (SPPC) and search phase (MCFHH), respectively. The F1 score (higher is better) is calculated as F1=2P∗R/(P+R), where P represents precision and R represents recall.

First, extensive experiments were conducted on six standard networks and three different linear SEMs to verify that our proposed algorithm is effective and robust. In our experiment, for each of the networks, we randomly sampled four datasets with 1000, 3000, 5000, and 10,000 cases. We report the mean and standard deviation of the evaluation indicators of 10 runs. Table 3, Table 4 and Table 5 present the results of the experiments on each dataset.

It can be seen in Table 3, Table 4 and Table 5 that for all the datasets, the standard deviations of the BIC, AD, DD, and RD are all 0 after multiple runs, indicating no variation in the results of the MCFHH algorithm across multiple runs. At the same time, the mean value of the BIC is consistently greater than that of the ABS across all datasets. The above results fully demonstrate the stable convergence performance of the MCFHH algorithm. Regardless of the size of the network, as long as a network with a higher score exists in the search space, the algorithm has the ability to find it. Regarding structural errors, we can observe that for datasets with structural errors in the output structure, the BIC surpassed the ABS (highlighted in bold in the table). The reason for this may be that the data cannot fully reflect the network structure’s characteristics. In addition, for all three SEMs, our algorithm yielded structures with stable F1 scores, which shows that the MCFHH algorithm is a robust DAG learning algorithm, whether applied to Gaussian or non-Gaussian models. In terms of execution time, RET increased very little and SET did not increase significantly as the sample size increased, indicating that our algorithm can handle large sample sizes. However, with the increase in the size of the network, SET increased much faster than RET. The reason for this is that the second stage search for working on the CSS increased the time cost. Next, we considered whether and under what circumstances the search space switching operator should be removed to save time. In theory, adding the search space switching operator can reduce the dependence of the algorithm on the sample size, which can also be seen in the insensitivity of each performance index to the sample size. Therefore, for small sample data, the search space switching operator may be an important guarantee for accuracy. Therefore, we report the performance of the MCFHH algorithm after removing the search space switching operator when the sample size was sufficient (10,000).

As shown in Table 6, for the four networks—alarm, win95pts, munin, and pigs—the same structure could still be output after deleting the search space switching operator. For the hepar2 and andes networks, although the same structure could not be output, the maximum coefficient of variation (standard deviation divided by the mean) of the output structure on the three SEMs was 0.08% and 0.04%, respectively. Therefore, when the sample size was sufficient, deleting the search space switching operator still reliably produced a high-score structure. However, for the relatively complex hepar2 and andes networks, it was difficult to guarantee the integrity of the GSS, even with a sufficient sample size, and the edges not covered by the GSS caused structural errors, which the search space switching operator aimed to correct by increasing the coverage of the search space. Regarding running time, Table 6 shows that except for the alarm and win95pts networks, the removal of the search space switching operator significantly reduced the time cost. For hepar2, munin, andes, and pigs, the average SET reduction rates were 50%, 84%, 72%, and 95%, respectively. In summary, in the first stage, the search space switching operator used the constraint method to limit the search space to improve search efficiency, and in the second stage, it corrected the structural errors caused by the incomplete search space by extending the coverage of the search space. In practice, we would likely face a trade-off between accuracy and speed.

### 5.3. Comparison with Other Algorithms

The performance of these comparison algorithms depends on the sample size. For a fair comparison, the sample size was uniformly set to 1000. Due to the serious impact of the search space switching operator on the performance of our algorithm, the algorithm that deletes the search space switching operator was also compared as a new algorithm, denoted as MCFHH1. Obviously, MCFHH1 represents the performance of our algorithm in the worst-case scenario.

Table 7 and Table 8 show the comparison results of the F1 scores and BIC scores, respectively, between our proposed algorithm and other algorithms in different SEMs. In these comparisons, MCFHH consistently outperformed the others (highlighted in bold in the table), which shows that our proposed algorithm is accurate and robust in linear SEMs. To further demonstrate the performance of our algorithm, we compared only MCFHH1 with other algorithms. The comparison of the BIC and F1 scores confirms the conclusion that MCFHH1>BNC-PSO>PCS>NOTEARS>PC>LiNGAM, which verifies that our algorithm maintains the reliability of the search even in the worst-case scenario.

Like the MCFHH1 algorithm, PCS also uses partial correlation to limit the search space. Its restrictions are more relaxed, so the coverage of its search space is wider. In theory, it is easier to search for a structure with a higher score. However, by comparing the BIC scores of PCS and MCFHH1, we found that the BIC scores of MCFHH1 were not lower than those of PCS on 12 datasets, most of which were concentrated on large-scale networks, such as munin, andes, and pigs. These results indicate that MCFHH1 has a stronger global search capability than PCS. Compared to MCFHH1, NOTEARS achieved higher BIC scores on 6 out of 18 datasets, while it failed to produce any output seven times. This means that NOTEARS is unstable and cannot stably output results. In addition, the performance of both the constraint-based method (PC-stable) and the exploiting structural asymmetries method (LiNGAM) was significantly worse compared to our method, especially on the andes network.

**Figure 2:**
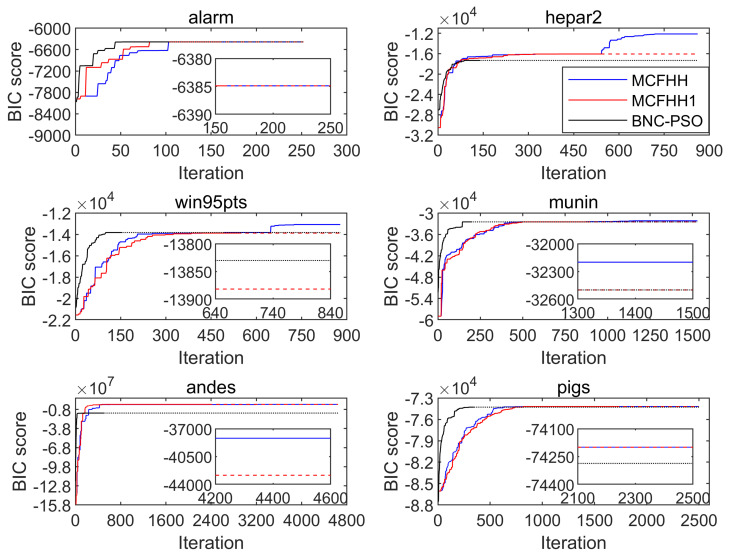
Convergence of the BIC scores of the three algorithms on the six networks.

Figure 2 illustrates the BIC scores with respect to the number of iterations for six networks, and the results after the algorithm stopped are indicated by dotted lines. As shown in Figure 2, three algorithms improved the quality of the solutions at the beginning of the search process, but BNC-PSO converged faster than MCFHH and MCFHH1. This phenomenon became more obvious on the last four networks at larger scales. With the increase in the number of iterations, the convergence speeds of the three algorithms tended to be the same. For the hepar2 and win95pts networks, we can clearly observe that the MCFHH algorithm continued to find structures with higher scores after the BNC-PSO and MCFHH1 algorithms converged. In addition, on the hepar2 and andes networks, the convergence accuracy of BNC-PSO was significantly lower than that of MCFHH and MCFHH1. This shows that the BNC-PSO algorithm cannot guarantee good performance in all cases. By comparing BNC-PSO and MCFHH1, we found that the latter achieved the highest BIC scores across all datasets and the highest F1 scores on 14 out of 18 datasets, with an order of magnitude difference in the BIC scores between the two on the andes network. Therefore, we can conclude that the latter has better generalizability and search capability than the former.

In summary, our algorithm increases population diversity by combining and optimizing a variety of nature-inspired heuristics, thereby increasing convergence accuracy and decreasing convergence speed. In our algorithm, the completeness of the search space guarantees convergence accuracy, but the complete search space greatly increases the time cost. Therefore, finding ways to limit the search space as much as possible while ensuring its completeness will be a direction for improving the performance of our algorithm. Overall, regardless of whether the data are Gaussian or non-Gaussian, our algorithm can stably output a structure that is closer to true causality. Our algorithm uses the constraint method to reduce the difficulty of the search method and uses the search method to correct the errors caused by the constraint method. To some extent, the advantages of the two types of methods are absorbed, and the defects of both methods are compensated for.

## 6. Conclusions and Future Research

In this paper, structural priors are obtained using the SPPC algorithm and integrated into the score search process to improve search efficiency. We prove the correctness and validity of the SPPC in theory. To make effective use of this prior knowledge, we devised a hyper-heuristic method called MCFHH to discover causality under linear SEMs. The experimental results show that the proposed method has better generalizability and search capability. Compared to state-of-the-art methods, it outputs structures that are closer to real causality. Additional efforts will be required to expand our work. In this paper, we have only proposed a hybrid approach under linear SEMs, and we intend to further investigate this hybrid method for both discrete and nonlinear problems. We will also develop better hyper-heuristic algorithms.

## Figures and Tables

**Table 1:** Summary of networks.

Network	Nodes	Edges	Max.indeg	Max.outdeg	Avg.deg
alarm	37	46	4	5	2.49
hepar2	70	123	6	17	3.51
win95pts	76	112	7	10	2.95
munin	189	282	3	15	2.98
andes	223	338	6	12	3.03
pigs	441	592	2	39	2.68

**Table 2:** Parameters of the MCFHH.

Param.	Value	Descriptions
*k*	0.01	The threshold of the Bayes factor
nPop	50	The population size
$L_{max}$	2·n	The maximum number of unpromoted iterations allowed
MaxIt	5000	The maximum number of iterations allowed
sn	5	The number of subgroups
μ	lnm	The threshold of pruning
$l_{m}$	20	Control parameters of the elimination and dispersal operator
zj	0.5	Percentage receiving expert guidance

**Table 3:** Performance of MCFHH algorithm on different datasets for SEM1. Bold denotes that BIC is greater than SBS.

Network	Dataset	BIC	SBS	AD	DD	RD	RET(s)	SET(s)	F1
alarm	1000	$-1.8710\;\times \;10^{4}$ ± 0	$-1.8710\;\times \;10^{4}$	0 ± 0	0 ± 0	0 ± 0	0.27	2.49	1 ± 0
	3000	$-5.5791\;\times \;10^{4}$ ± 0	$-5.5791\;\times \;10^{4}$	0 ± 0	0 ± 0	0 ± 0	0.28	2.98	1 ± 0
	5000	$-9.2964\;\times \;10^{4}$ ± 0	$-9.2964\;\times \;10^{4}$	0 ± 0	0 ± 0	0 ± 0	0.29	5.11	1 ± 0
	10,000	$-1.8598\;\times \;10^{5}$ ± 0	$-1.8598\;\times \;10^{5}$	0 ± 0	0 ± 0	0 ± 0	0.29	9.49	1 ± 0
hepar2	1000	$-3.5372\;\times \;10^{4}$ ± 0	$-3.5372\;\times \;10^{4}$	0 ± 0	0 ± 0	0 ± 0	1.08	28.07	1 ± 0
	3000	$-1.0568\;\times \;10^{5}$ ± 0	$-1.0568\;\times \;10^{5}$	0 ± 0	0 ± 0	0 ± 0	1.74	35.54	1 ± 0
	5000	$-1.7600\;\times \;10^{5}$ ± 0	$-1.7600\;\times \;10^{5}$	0 ± 0	0 ± 0	0 ± 0	1.99	69.39	1 ± 0
	10,000	$-3.5157\;\times \;10^{5}$ ± 0	$-3.5157\;\times \;10^{5}$	0 ± 0	0 ± 0	0 ± 0	2.06	151.38	1 ± 0
win95pts	1000	$-3.8374\;\times \;10^{4}$ ± 0	$-3.8374\;\times \;10^{4}$	0 ± 0	0 ± 0	0 ± 0	1.82	19.16	1 ± 0
	3000	$-1.1480\;\times \;10^{5}$ ± 0	$-1.1480\;\times \;10^{5}$	0 ± 0	0 ± 0	0 ± 0	2.24	19.98	1 ± 0
	5000	$-1.9122\;\times \;10^{5}$ ± 0	$-1.9122\;\times \;10^{5}$	0 ± 0	0 ± 0	0 ± 0	2.62	39.27	1 ± 0
	10,000	$-3.8166\;\times \;10^{5}$ ± 0	$-3.8166\;\times \;10^{5}$	0 ± 0	0 ± 0	0 ± 0	2.67	65.02	1 ± 0
munin	1000	**−9.5488**$\;\times \;10^{4}$ ± 0	$-9.5663\;\times \;10^{4}$	0 ± 0	59 ± 0	0 ± 0	1.62	160.46	0.8832 ± 0
	3000	**−2.8535**$\;\times \;10^{5}$ ± 0	$-2.8556\;\times \;10^{5}$	0 ± 0	59 ± 0	0 ± 0	1.74	172.43	0.8832 ± 0
	5000	**−4.7464**$\;\times \;10^{5}$ ± 0	$-4.7486\;\times \;10^{5}$	0 ± 0	59 ± 0	0 ± 0	1.82	272.54	0.8832 ± 0
	10,000	**−9.4648**$\;\times \;10^{5}$ ± 0	$-9.4673\;\times \;10^{5}$	0 ± 0	59 ± 0	0 ± 0	1.96	518.02	0.8832 ± 0
andes	1000	$-1.1296\;\times \;10^{5}$ ± 0	$-1.1296\;\times \;10^{5}$	0 ± 0	0 ± 0	0 ± 0	4.92	660.71	1 ± 0
	3000	$-3.3678\;\times \;10^{5}$ ± 0	$-3.3678\;\times \;10^{5}$	0 ± 0	0 ± 0	0 ± 0	5.69	617.39	1 ± 0
	5000	$-5.6010\;\times \;10^{5}$ ± 0	$-5.6010\;\times \;10^{5}$	0 ± 0	0 ± 0	0 ± 0	6.19	875.83	1 ± 0
	10,000	$-1.1172\;\times \;10^{6}$ ± 0	$-1.1172\;\times \;10^{6}$	0 ± 0	0 ± 0	0 ± 0	6.21	1640.18	1 ± 0
pigs	1000	**−2.2197**$\;\times \;10^{5}$ ± 0	$-2.2301\;\times \;10^{5}$	0 ± 0	357 ± 0	0 ± 0	6.90	2850.50	0.5683 ± 0
	3000	**−6.6343**$\;\times \;10^{5}$ ± 0	$-6.6467\;\times \;10^{5}$	0 ± 0	357 ± 0	0 ± 0	6.93	2635.21	0.5683 ± 0
	5000	**−1.1037**$\;\times \;10^{6}$ ± 0	$-1.1051\;\times \;10^{6}$	0 ± 0	357 ± 0	0 ± 0	6.79	2914.08	0.5683 ± 0
	10,000	**−2.2062**$\;\times \;10^{6}$ ± 0	$-2.2077\;\times \;10^{6}$	0 ± 0	357 ± 0	0 ± 0	6.93	3388.36	0.5683 ± 0

**Table 4:** Performance of MCFHH algorithm on different datasets for SEM2. Bold denotes that BIC is greater than SBS.

Network	Dataset	BIC	SBS	AD	DD	RD	RET(s)	SET(s)	F1
alarm	1000	$-1.8638\;\times \;10^{4}$ ± 0	$-1.8638\;\times \;10^{4}$	0 ± 0	0 ± 0	0 ± 0	0.20	2.94	1 ± 0
	3000	$-5.5744\;\times \;10^{4}$ ± 0	$-5.5744\;\times \;10^{4}$	0 ± 0	0 ± 0	0 ± 0	0.26	2.84	1 ± 0
	5000	$-9.2944\;\times \;10^{4}$ ± 0	$-9.2944\;\times \;10^{4}$	0 ± 0	0 ± 0	0 ± 0	0.30	5.97	1 ± 0
	10,000	$-1.8587\;\times \;10^{5}$ ± 0	$-1.8587\;\times \;10^{5}$	0 ± 0	0 ± 0	0 ± 0	0.28	7.45	1 ± 0
hepar2	1000	$-3.5318\;\times \;10^{4}$ ± 0	$-3.5318\;\times \;10^{4}$	0 ± 0	0 ± 0	0 ± 0	1.23	20.57	1 ± 0
	3000	$-1.0562\;\times \;10^{5}$ ± 0	$-1.0562\;\times \;10^{5}$	0 ± 0	0 ± 0	0 ± 0	2.18	23.88	1 ± 0
	5000	$-1.7604\;\times \;10^{5}$ ± 0	$-1.7604\;\times \;10^{5}$	0 ± 0	0 ± 0	0 ± 0	2.51	47.62	1 ± 0
	10,000	$-3.5157\;\times \;10^{5}$ ± 0	$-3.5157\;\times \;10^{5}$	0 ± 0	0 ± 0	0 ± 0	2.72	97.38	1 ± 0
win95pts	1000	**−3.8309**$\;\times \;10^{4}$ ± 0	$-3.8310\;\times \;10^{4}$	0 ± 0	0 ± 0	1 ± 0	1.62	14.12	0.9911 ± 0
	3000	$-1.1476\;\times \;10^{5}$ ± 0	$-1.1476\;\times \;10^{5}$	0 ± 0	0 ± 0	0 ± 0	2.18	17.18	1 ± 0
	5000	$-1.9119\;\times \;10^{5}$ ± 0	$-1.9119\;\times \;10^{5}$	0 ± 0	0 ± 0	0 ± 0	2.39	34.61	1 ± 0
	10,000	$-3.8160\;\times \;10^{5}$ ± 0	$-3.8160\;\times \;10^{5}$	0 ± 0	0 ± 0	0 ± 0	2.49	60.28	1 ± 0
munin	1000	**−9.5380**$\;\times \;10^{4}$ ± 0	$-9.5561\;\times \;10^{4}$	0 ± 0	59 ± 0	1 ± 0	1.59	179.94	0.8792 ± 0
	3000	**−2.8533**$\;\times \;10^{5}$ ± 0	$-2.8554\;\times \;10^{5}$	0 ± 0	59 ± 0	0 ± 0	1.76	140.15	0.8832 ± 0
	5000	**−4.7471**$\;\times \;10^{5}$ ± 0	$-4.7495\;\times \;10^{5}$	0 ± 0	59 ± 0	0 ± 0	1.79	241.23	0.8832 ± 0
	10,000	**−9.4647**$\;\times \;10^{5}$ ± 0	$-9.4673\;\times \;10^{5}$	0 ± 0	59 ± 0	0 ± 0	1.86	284.04	0.8832 ± 0
andes	1000	**−1.1280**$\;\times \;10^{5}$ ± 0	$-1.1281\;\times \;10^{5}$	1 ± 0	0 ± 0	0 ± 0	6.51	559.27	0.9985 ± 0
	3000	$-3.3677\;\times \;10^{5}$ ± 0	$-3.3677\;\times \;10^{5}$	0 ± 0	0 ± 0	0 ± 0	5.93	552.35	1 ± 0
	5000	$-5.6012\;\times \;10^{5}$ ± 0	$-5.6012\;\times \;10^{5}$	0 ± 0	0 ± 0	0 ± 0	5.33	582.98	1 ± 0
	10,000	$-1.1171\;\times \;10^{6}$ ± 0	$-1.1171\;\times \;10^{6}$	0 ± 0	0 ± 0	0 ± 0	4.19	931.86	1 ± 0
pigs	1000	**−2.2209**$\;\times \;10^{5}$ ± 0	$-2.2316\;\times \;10^{5}$	1 ± 0	357 ± 0	0 ± 0	6.90	3184.71	0.5676 ± 0
	3000	**−6.6328**$\;\times \;10^{5}$ ± 0	$-6.6455\;\times \;10^{5}$	0 ± 0	357 ± 0	1 ± 0	6.78	2807.74	0.5659 ± 0
	5000	**−1.1040**$\;\times \;10^{6}$ ± 0	$-1.1053\;\times \;10^{6}$	1 ± 0	357 ± 0	0 ± 0	6.72	3399.62	0.5676 ± 0
	10,000	**−2.2065**$\;\times \;10^{6}$ ± 0	$-2.2079\;\times \;10^{6}$	0 ± 0	357 ± 0	0 ± 0	6.75	3187.43	0.5683 ± 0

**Table 5:** Performance of MCFHH algorithm on different datasets for SEM3. Bold denotes that BIC is greater than SBS.

Network	Dataset	BIC	SBS	AD	DD	RD	RET(s)	SET(s)	F1
alarm	1000	$-6.3849\;\times \;10^{3}$ ± 0	$-6.3849\;\times \;10^{3}$	0 ± 0	0 ± 0	0 ± 0	0.30	1.95	1 ± 0
	3000	$-1.8688\;\times \;10^{4}$ ± 0	$-1.8688\;\times \;10^{4}$	0 ± 0	0 ± 0	0 ± 0	0.32	2.57	1 ± 0
	5000	$-3.1042\;\times \;10^{4}$ ± 0	$-3.1042\;\times \;10^{4}$	0 ± 0	0 ± 0	0 ± 0	0.26	5.51	1 ± 0
	10,000	$-6.1808\;\times \;10^{4}$ ± 0	$-6.1808\;\times \;10^{4}$	0 ± 0	0 ± 0	0 ± 0	0.30	8.12	1 ± 0
hepar2	1000	$-1.2133\;\times \;10^{4}$ ± 0	$-1.2133\;\times \;10^{4}$	0 ± 0	0 ± 0	0 ± 0	1.01	23.25	1 ± 0
	3000	$-3.5491\;\times \;10^{4}$ ± 0	$-3.5491\;\times \;10^{4}$	0 ± 0	0 ± 0	0 ± 0	1.49	28.99	1 ± 0
	5000	$-5.8769\;\times \;10^{4}$ ± 0	$-5.8769\;\times \;10^{4}$	0 ± 0	0 ± 0	0 ± 0	1.70	52.07	1 ± 0
	10,000	$-1.1715\;\times \;10^{5}$ ± 0	$-1.1715\;\times \;10^{5}$	0 ± 0	0 ± 0	0 ± 0	2.03	129.98	1 ± 0
win95pts	1000	$-1.3083\;\times \;10^{4}$ ± 0	$-1.3083\;\times \;10^{4}$	0 ± 0	0 ± 0	0 ± 0	1.68	18.61	1 ± 0
	3000	$-3.8459\;\times \;10^{4}$ ± 0	$-3.8459\;\times \;10^{4}$	0 ± 0	0 ± 0	0 ± 0	2.09	24.80	1 ± 0
	5000	$-6.3749\;\times \;10^{4}$ ± 0	$-6.3749\;\times \;10^{4}$	0 ± 0	0 ± 0	0 ± 0	2.26	43.62	1 ± 0
	10,000	$-1.2713\;\times \;10^{5}$ ± 0	$-1.2713\;\times \;10^{5}$	0 ± 0	0 ± 0	0 ± 0	2.39	74.95	1 ± 0
munin	1000	**−3.2199**$\;\times \;10^{4}$ ± 0	$-3.2393\;\times \;10^{4}$	0 ± 0	59 ± 0	0 ± 0	1.64	177.87	0.8832 ± 0
	3000	**−9.5189**$\;\times \;10^{4}$ ± 0	$-9.5418\;\times \;10^{4}$	0 ± 0	59 ± 0	0 ± 0	1.77	156.88	0.8832 ± 0
	5000	**−1.5820**$\;\times \;10^{5}$ ± 0	$-1.5844\;\times \;10^{5}$	0 ± 0	59 ± 0	0 ± 0	1.80	248.07	0.8832 ± 0
	10,000	**−3.1594**$\;\times \;10^{5}$ ± 0	$-3.1620\;\times \;10^{5}$	0 ± 0	59 ± 0	0 ± 0	1.83	415.10	0.8832 ± 0
andes	1000	$-3.8190\;\times \;10^{4}$ ± 0	$-3.8190\;\times \;10^{4}$	0 ± 0	0 ± 0	0 ± 0	5.87	676.99	1 ± 0
	3000	$-1.1267\;\times \;10^{5}$ ± 0	$-1.1267\;\times \;10^{5}$	0 ± 0	0 ± 0	0 ± 0	5.54	647.91	1 ± 0
	5000	$-1.8691\;\times \;10^{5}$ ± 0	$-1.8691\;\times \;10^{5}$	0 ± 0	0 ± 0	0 ± 0	5.37	947.98	1 ± 0
	10,000	$-3.7329\;\times \;10^{5}$ ± 0	$-3.7329\;\times \;10^{5}$	0 ± 0	0 ± 0	0 ± 0	4.48	1733.18	1 ± 0
pigs	1000	**−7.4199**$\;\times \;10^{4}$ ± 0	$-7.5375\;\times \;10^{4}$	0 ± 0	357 ± 0	0 ± 0	6.96	2984.92	0.5683 ± 0
	3000	**−2.2124**$\;\times \;10^{5}$ ± 0	$-2.2261\;\times \;10^{5}$	0 ± 0	357 ± 0	0 ± 0	6.79	3229.69	0.5683 ± 0
	5000	**−3.6861**$\;\times \;10^{5}$ ± 0	$-3.7007\;\times \;10^{5}$	0 ± 0	357 ± 0	0 ± 0	6.74	3335.36	0.5683 ± 0
	10,000	**−7.3672**$\;\times \;10^{5}$ ± 0	$-7.3831\;\times \;10^{5}$	0 ± 0	357 ± 0	0 ± 0	6.75	3452.05	0.5683 ± 0

**Table 6:** Performance of MCFHH algorithm without the switching operator.

Network	SEM	BIC	SBS	AD	DD	RD	RET(s)	SET(s)	F1
alarm	1	$-1.8598\;\times \;10^{5}$ ± 0	$-1.8598\;\times \;10^{5}$	0 ± 0	0 ± 0	0 ± 0	0.27	2.88	1 ± 0
	2	$-1.8587\;\times \;10^{5}$ ± 0	$-1.8587\;\times \;10^{5}$	0 ± 0	0 ± 0	0 ± 0	0.28	2.37	1 ± 0
	3	$-6.1808\;\times \;10^{4}$ ± 0	$-6.1808\;\times \;10^{4}$	0 ± 0	0 ± 0	0 ± 0	0.29	2.73	1 ± 0
hepar2	1	$-3.7076\;\times \;10^{5}$ ± 290.56	$-3.5157\;\times \;10^{5}$	0.50 ± 1.58	2 ± 0	1.60 ± 1.90	1.08	12.56	0.9768 ± 0.0215
	2	$-3.5297\;\times \;10^{5}$ ± 0	$-3.5157\;\times \;10^{5}$	0 ± 0	1 ± 0	0 ± 0	1.74	10.77	0.9959 ± 0
	3	$-1.2365\;\times \;10^{5}$ ± 0	$-1.1715\;\times \;10^{5}$	0 ± 0	2 ± 0	1 ± 0	1.99	12.17	0.9836 ± 0
win95pts	1	$-3.8166\;\times \;10^{5}$ ± 0	$-3.8166\;\times \;10^{5}$	0 ± 0	0 ± 0	0 ± 0	1.82	13.22	1 ± 0
	2	$-3.8160\;\times \;10^{5}$ ± 0	$-3.8160\;\times \;10^{5}$	0 ± 0	0 ± 0	0 ± 0	2.24	12.36	1 ± 0
	3	$-1.2713\;\times \;10^{5}$ ± 0	$-1.2713\;\times \;10^{5}$	0 ± 0	0 ± 0	0 ± 0	2.62	14.57	1 ± 0
munin	1	$-9.4648\;\times \;10^{5}$ ± 0	$-9.4673\;\times \;10^{5}$	0 ± 0	59 ± 0	0 ± 0	1.62	28.46	0.8832 ± 0
	2	$-9.4647\;\times \;10^{5}$ ± 0	$-9.4671\;\times \;10^{5}$	0 ± 0	59 ± 0	0 ± 0	1.74	25.07	0.8832 ± 0
	3	$-3.1594\;\times \;10^{5}$ ± 0	$-3.1620\;\times \;10^{5}$	0 ± 0	59 ± 0	0 ± 0	1.82	29.70	0.8832 ± 0
andes	1	$-1.1223\;\times \;10^{6}$ ± 0	$-1.1172\;\times \;10^{6}$	5 ± 0	1 ± 0	1 ± 0	4.92	206.39	0.9882 ± 0
	2	$-1.1204\;\times \;10^{6}$ ± 40.23	$-1.1171\;\times \;10^{6}$	1.90 ± 0.32	1 ± 0	1 ± 0	5.69	105.17	0.9928 ± 0.0005
	3	$-3.7807\;\times \;10^{5}$ ± 139.81	$-3.7329\;\times \;10^{5}$	7 ± 0.47	2 ± 0	2.10 ± 0.32	6.19	232.26	0.9806 ± 0.0015
pigs	1	$-2.2062\;\times \;10^{6}$ ± 0	$-2.2077\;\times \;10^{6}$	0 ± 0	357 ± 0	0 ± 0	6.90	158.35	0.5683 ± 0
	2	$-2.2065\;\times \;10^{6}$ ± 0	$-2.2079\;\times \;10^{6}$	0 ± 0	357 ± 0	0 ± 0	6.93	163.47	0.5683 ± 0
	3	$-7.3672\;\times \;10^{5}$ ± 0	$-7.3831\;\times \;10^{5}$	0 ± 0	357 ± 0	0 ± 0	6.79	156.06	0.5683 ± 0

**Table 7:** F1 scores of the algorithms for different SEMs. Bold denotes the F1 score that was the best found amongst all methods. “-” indicates that no result is displayed.

SEM	Network	PC-Stable	LiNGAM	PCS	NOTEARS	BNC-PSO	MCFHH1	MCFHH
SEM1	alarm	0.8539	0.6387	0.9787	0.9892	0.9892	**1**	**1**
	hepar2	0.5871	0.7103	0.8593	-	0.9049	0.9153	**1**
	win95pts	0.7882	0.4683	0.8631	0.9912	0.9715	0.9740	**1**
	munin	0.7546	0.3350	0.6314	-	0.8611	0.8720	**0.8832**
	andes	0.5497	0.2823	0.6826	-	0.8768	0.9014	**1**
	pigs	0.4766	0.1650	0.3612	**0.5683**	0.5272	**0.5683**	**0.5683**
SEM2	alarm	0.8605	0.6116	0.9787	0.9451	0.9778	0.9778	**1**
	hepar2	0.6346	0.7117	0.8651	0.7603	0.9048	0.8996	**1**
	win95pts	0.8491	0.6154	0.9136	0.9646	0.9695	0.9683	**0.9911**
	munin	0.7976	0.3724	0.6216	0.8151	0.8607	0.8652	**0.8792**
	andes	0.8394	0.4008	0.6000	-	0.9327	0.9327	**0.9985**
	pigs	0.4174	0.1499	0.3265	0.5600	0.5141	**0.5676**	**0.5676**
SEM3	alarm	0.8276	0.9388	0.9892	0.9053	**1**	**1**	**1**
	hepar2	0.5545	0.7413	0.8696	-	0.9392	0.9180	**1**
	win95pts	0.8020	0.5542	0.9912	0.9442	0.9713	0.9691	**1**
	munin	0.7670	0.3627	0.7876	-	0.8739	0.8743	**0.8832**
	andes	0.5209	0.2649	0.7862	-	0.9200	0.9423	**1**
	pigs	0.4687	0.1783	0.5098	0.5642	0.5631	**0.5683**	**0.5683**

**Table 8:** BIC scores of the algorithms for different SEMs. Bold denotes the BIC score that was the best found amongst all methods. “-” indicates that no result is displayed.

SEM	Network	PC-Stable	LiNGAM	PCS	NOTEARS	BNC-PSO	MCFHH1	MCFHH
SEM1	alarm	$-2.23\;\times \;10^{4}$	$-2.15\;\times \;10^{4}$	$-1.87\;\times \;10^{4}$	$-1.87\;\times \;10^{4}$	**−1.87** $\;\times \;10^{4}$	**−1.87** $\;\times \;10^{4}$	**−1.87** $\;\times \;10^{4}$
	hepar2	$-1.48\;\times \;10^{5}$	$-4.26\;\times \;10^{4}$	$-3.97\;\times \;10^{4}$	-	$-4.73\;\times \;10^{4}$	$-4.57\;\times \;10^{4}$	**−3.54** $\;\times \;10^{4}$
	win95pts	$-7.70\;\times \;10^{4}$	$-4.94\;\times \;10^{4}$	$-4.09\;\times \;10^{4}$	$-3.84\;\times \;10^{4}$	$-4.08\;\times \;10^{4}$	$-4.08\;\times \;10^{4}$	**−3.84** $\;\times \;10^{4}$
	munin	$-1.29\;\times \;10^{5}$	$-1.51\;\times \;10^{5}$	$-1.22\;\times \;10^{5}$	-	$-9.86\;\times \;10^{4}$	$-9.76\;\times \;10^{4}$	**−9.55** $\;\times \;10^{4}$
	andes	$-3.97\;\times \;10^{8}$	$-5.77\;\times \;10^{8}$	$-4.39\;\times \;10^{5}$	-	$-6.49\;\times \;10^{6}$	$-2.35\;\times \;10^{5}$	**−1.13** $\;\times \;10^{5}$
	pigs	$-2.28\;\times \;10^{5}$	$-2.46\;\times \;10^{5}$	$-2.26\;\times \;10^{5}$	**−2.22** $\;\times \;10^{5}$	$-2.23\;\times \;10^{5}$	**−2.22** $\;\times \;10^{5}$	**−2.22** $\;\times \;10^{5}$
SEM2	alarm	$-2.04\;\times \;10^{4}$	$-1.95\;\times \;10^{4}$	$-1.86\;\times \;10^{4}$	$-1.88\;\times \;10^{4}$	$-1.90\;\times \;10^{4}$	$-1.90\;\times \;10^{4}$	**−1.86** $\;\times \;10^{4}$
	hepar2	$-4.48\;\times \;10^{4}$	$-3.79\;\times \;10^{4}$	$-3.63\;\times \;10^{4}$	$-3.68\;\times \;10^{4}$	$-3.69\;\times \;10^{4}$	$-3.69\;\times \;10^{4}$	**−3.53** $\;\times \;10^{4}$
	win95pts	$-4.63\;\times \;10^{4}$	$-4.03\;\times \;10^{4}$	$-3.83\;\times \;10^{4}$	$-3.84\;\times \;10^{4}$	$-3.85\;\times \;10^{4}$	$-3.85\;\times \;10^{4}$	**−3.83** $\;\times \;10^{4}$
	munin	$-9.70\;\times \;10^{4}$	$-1.06\;\times \;10^{5}$	$-9.85\;\times \;10^{4}$	$-9.59\;\times \;10^{4}$	$-9.57\;\times \;10^{4}$	$-9.56\;\times \;10^{4}$	**−9.54** $\;\times \;10^{4}$
	andes	$-1.52\;\times \;10^{5}$	$-1.47\;\times \;10^{5}$	$-1.24\;\times \;10^{5}$	-	$-1.18\;\times \;10^{5}$	$-1.16\;\times \;10^{5}$	**−1.13** $\;\times \;10^{5}$
	pigs	$-2.25\;\times \;10^{5}$	$-2.32\;\times \;10^{5}$	$-2.23\;\times \;10^{5}$	$-2.22\;\times \;10^{5}$	$-2.22\;\times \;10^{5}$	**−2.22** $\;\times \;10^{5}$	**−2.22** $\;\times \;10^{5}$
SEM3	alarm	$-7.87\;\times \;10^{3}$	$-6.40\;\times \;10^{3}$	$-6.39\;\times \;10^{3}$	$-6.50\;\times \;10^{3}$	**−6.38** $\;\times \;10^{3}$	**−6.38** $\;\times \;10^{3}$	**−6.38** $\;\times \;10^{3}$
	hepar2	$-3.90\;\times \;10^{4}$	$-1.57\;\times \;10^{4}$	$-1.38\;\times \;10^{4}$	-	$-1.69\;\times \;10^{4}$	$-1.61\;\times \;10^{4}$	**−1.21** $\;\times \;10^{4}$
	win95pts	$-2.07\;\times \;10^{4}$	$-1.61\;\times \;10^{4}$	$-1.31\;\times \;10^{4}$	$-1.32\;\times \;10^{4}$	$-1.39\;\times \;10^{4}$	$-1.39\;\times \;10^{4}$	**−1.31** $\;\times \;10^{4}$
	munin	$-4.68\;\times \;10^{4}$	$-4.28\;\times \;10^{4}$	$-3.37\;\times \;10^{4}$	-	$-3.25\;\times \;10^{4}$	$-3.25\;\times \;10^{4}$	**−3.22** $\;\times \;10^{4}$
	andes	$-1.88\;\times \;10^{8}$	$-6.22\;\times \;10^{7}$	$-1.04\;\times \;10^{5}$	-	$-2.35\;\times \;10^{6}$	$-4.26\;\times \;10^{4}$	**−3.82** $\;\times \;10^{4}$
	pigs	$-7.60\;\times \;10^{4}$	$-8.31\;\times \;10^{4}$	$-7.55\;\times \;10^{4}$	$-7.42\;\times \;10^{4}$	$-7.44\;\times \;10^{4}$	**−7.42** $\;\times \;10^{4}$	**−7.42** $\;\times \;10^{4}$

## Data Availability

The true networks of all eight datasets are known, and they are publicly available (http://www.bnlearn.com/bnrepository, accessed on 10 May 2024).

## Abbreviations

The following abbreviations are used in this manuscript:

ABC	Artificial bee colony
BF	Bayes factor
BFO	Bacterial foraging optimization
BIC	Bayesian information criterion
CI	Conditional independence
CSS	Complete search space
DAG	Directed acyclic graph
GSS	Global search space
LLH	Low-level heuristics
LSS	Local search space
MB	Markov blanket
MCFHH	Multi-population choice function hyper-heuristic
NLL	Negative log-likelihood
OSP	Open simple path
PSO	Particle swarm optimization
SEM	Structural equation model
SPPC	Structural priors by partial correlation
